# Chromophore Deprotonation State Alters the Optical Properties of Blue Chromoprotein

**DOI:** 10.1371/journal.pone.0134108

**Published:** 2015-07-28

**Authors:** Cheng-Yi Chiang, Cheng-Chung Lee, Shin-Yi Lo, Andrew H.-J. Wang, Huai-Jen Tsai

**Affiliations:** 1 Institute of Molecular and Cellular Biology, National Taiwan University, Taipei, Taiwan; 2 Institute of Biological Chemistry, Academia Sinica, Taipei, Taiwan; 3 Core Facility for Protein Production and X-ray Structural Analysis, Academia Sinica, Taipei, Taiwan; 4 Institute of Biomedical Sciences, MacKay Medical College, New Taipei City, Taiwan; Australian National University, AUSTRALIA

## Abstract

Chromoproteins (CPs) have unique colors and can be used in biological applications. In this work, a novel blue CP with a maximum absorption peak (λ_max_) at 608 nm was identified from the carpet anemone *Stichodactyla gigantea* (sgBP). *In vivo* expression of sgBP in zebrafish would change the appearance of the fishes to have a blue color, indicating the potential biomarker function. To enhance the color properties, the crystal structure of sgBP at 2.25 Å resolution was determined to allow structure-based protein engineering. Among the mutations conducted in the Gln-Tyr-Gly chromophore and chromophore environment, a S157C mutation shifted the λ_max_ to 604 nm with an extinction coefficient (ε) of 58,029 M^-1^·cm^-1^ and darkened the blue color expression. The S157C mutation in the sgBP chromophore environment could affect the color expression by altering the deprotonation state of the phenolic group in the chromophore. Our results provide a structural basis for the blue color enhancement of the biomarker development.

## Introduction

Naturally existing anthozoan chromoproteins (CPs) have vivid colors and can be used to develop fluorescent proteins (FPs) [1] such as the far-red HcRed and photoswitchable KFP [2–3]. The first characterized CP from marine organisms is the pocilloporin isolated from the *Pocillopora damicornis*, which has a high absorbance of light at wavelengths of 390 nm and 560~590 nm, resulting in a pink appearance [4]. CPs also exist in other anemones or corals, e.g., the purple hcCP, the purple-red asCP, the purple-blue cjCP in *Heteractis crispa*, *Anemonia sulcata*, and *Goniopora tenuidens*, respectively [5–6]. Compared to purple CPs, only a few blue chromoproteins (BCPs) have been characterized: the Rtms5 belonging to the pocilloporin family, the aeCP597 from *Actinia equina*, and the cjBlue from *Cnidopus japonicus* [7–9]. Some sea anemones that belong to the *Stichodactyla* species have a naturally blue look, and these sea anemones could be new sources to identify new BCPs.

As a member of GFP-like protein family, CPs have a barrel-shaped monomer structure embedded with a chromophore, and each protein monomer can assemble into dimer or tetramer structures [10–12]. The chromophore responsible for the optical properties in GFP-like proteins is composed of X-Tyr-Gly and is able to form an imidazolinone structure through an autocyclization step [13–14], which involves the residues in the vicinity of the chromophore environment such as the highly conserved E215 and R95 in DsRed and the E222 and R96 in avGFP [15–16]. The imidazolinone structure, together with the phenolic group in the chromophore, forms a conjugated system of π-electrons that is regarded as the cause of the excitation and emission in GFP-like proteins.

Based on the *cis/trans*-conformation and the planarity of the chromophore, GFP-like proteins can be categorized. FPs usually adopt coplanar conformation, no matter what they are *cis* or *trans* orientation [17–21], while the non-planarity results in the non-fluorescent property of CPs [22]. For instance, the blue CP Rtms5 adopts a *trans* non-coplanar form with a Gln-Tyr-Gly chromophore composition identical to DsRed [7].

Because of the long absorption wavelength, the appearance of BCP is easily affected by the transgenic organisms. Lights with high energy are needed for exciting the proteins with a blue emission, and the high energy light might be harmful to organisms. Therefore, enhancing the blue color of BCP is required for biomarker development. In this work, a new BCP named sgBP was isolated from *Stichodactyla gigantea*, the giant carpet anemone. To investigate the blue color-determining mechanism of sgBP, spectrometry was used to define the optical properties of sgBP, and X-ray crystallography was used to determine the sgBP structure. According to the optical properties and crystal structure of sgBP, structure-based, site-directed mutagenesis occurred. The results in this study showed that the single mutation at S157 of sgBP could alter the deprotonation state of the chromophore and lead to a blueshifted maximum absorption peak in the sgBP absorption spectrum.

## Materials and Methods

### Ethics statement

The National Taiwan University Institutional Animal Care and Use Committee (IACUC) reviewed and approved the protocol described below (NTU-102-EL-13). No specific ethics approval was required for this project, as all zebrafish (*Danio rerio*) used in this study were between 0 and 3 days post-fertilization (dpf). This procedure is not considered painful because embryos at this early stage have no pain perception.

### Isolation of wild-type sgBP from *S*. *gigantea*

Samples of *S*. *gigantea* originating from the Indo-Pacific were purchased from local aquaria. The tentacles were cut into small pieces and ground into powder using liquid nitrogen. The sample powder was collected in a 1.5-ml tube and mixed with 0.15 g acid-washed glass beads (Sigma-Aldrich), 200 μl PBS buffer and 0.2 M phenylmethanesulfonyl fluoride (Sigma-Aldrich, 1 μl) as a protease inhibitor. The sample solution was vibrated at 4°C for 2 min. Five repetitions were carried out before the homogenized protein sample was collected. The protein extract was centrifuged at 16,000 x g for 30 min at 4°C, and the supernatant was collected. The CP-containing supernatant was further analyzed by anion exchange and gel filtration using fast protein liquid chromatography (FPLC) to isolate the CP fraction. The CP fraction was analyzed on 12% SDS gel at 80 V for 20 min on 4% stacking gel and at 120 V for 100 min on 12% separating gel using a Bio-Rad Mini-PROTEAN Tetra Cell system. The separated 25 kDa protein fractions were electrotransferred under 100 V, 400 mA for 60 min onto a PVDF membrane. The CP major band on the membrane was cut and then digested by trypsin (Promega). Liquid chromatography-tandem mass spectrometry (LC-MS/MS) analysis was then performed on the trypsin-digested CP.

### Synthesis of sgBP cDNA

Total RNAs of *S*. *gigantea* were isolated using a TRIzol reagent kit (Ambion). Synthesis and amplification of cDNA were performed using a Superscript III cDNA synthesis kit (Invitrogen). During the cDNA synthesis, dT(15)-T7 primer (AAACGACGGCCAGTGAATTTAATACGACTCACTATAGGCGCTTTTTTTTTTTTTTTT) and TS primer (AAGCAGTGGTAACAACGCAGAGTACGCGGG) were added to the 3’- and 5’-end of the cDNAs as the template switching sequences. The degenerate primer sgBP_deg (TGYGGNCARTCNTTRATGGC, R = A or G, Y = C or T, N = A, T, C or G) was designed according to the partial peptide sequences, CGQSLMA, obtained from LC-MS/MS (S1 Fig). The 3’-end of the sgBP cDNA was determined by 3’-Rapid Amplification of the cDNA Ends (RACE) using the sgBP_deg and dT(15)-T7 primers. Design of the sgBP_3’*Xho*I primer (CTCGAGTTAGTGATGTCCAAGCTTTGATGG) was based on the 3’-end sequence of sgBP cDNA. The 5’-end of sgBP cDNA was determined by 5’-RACE using primers of sgBP_3’*Xho*I and TS, and the sgBP_5’*Nde*I primer (CATATGGCTTCATTGGTTAAGAAGGATATG) was designed according to the 5’-end of the sgBP cDNA. By combining the sgBP-5’*Nde*I primer and the sgBP_3’*Xho*I primer, we amplified the sgBP cDNA with *Nde*I and *Xho*I cutting sites.

### Expression of sgBP in zebrafish embryos

To generate plasmid expression in zebrafish embryos, the DNA sequences of sgBP were used as a template to perform PCR using a sgBP_KozakAgeI primer (ACCGGTCGCC ACCATGGTTGCCATCCCAGAAAAC) and a sgBP_XhoIinj primer (CTCGAGTTAATTGTGACCAAGTTTAGATGGTACAG). The sgBP fragment containing *Age*I and *Xho*I cutting sites was constructed into pGEM-T Easy Vector (Promega) by TA cloning, generating the plasmid pGEM-sgBP-A/X. This plasmid was treated with *Age*I (NEB) and *Xho*I (NEB) to obtain the sgBP fragments with *Age*I and *Xho*I sticky ends. Plasmid containing a zebrafish α-actin promoter [23] was digested with *Age*I and *Xho*I. The resultant plasmid was then ligated with the sgBP fragment to generate the pZα-sgBP plasmid. The plasmid pZα-sgBP was linearized by *Not*I (NEB) and then mixed with injection dye (phenol red) and sterilized water to a concentration of 25 ng/μl. The linearized expression plasmids were microinjected into one-celled zebrafish embryos.

### Protein production

To generate recombinant sgBP and its derivatives for characterization, spectrometry measurement and crystallization, the sgBP and its mutated cDNAs with *Nde*I and *Xho*I ends were constructed into the expression vector pET-15b. The 5’- and 3’- ends of the mutated sequences were put together with sgBP_5’*Nde*I and sgBP_3’*Bam*HI primers to perform PCR for amplification of the full-length mutated sequences. The full-length mutated DNA sequences were constructed into pGEM-T Easy Vector (Promega) by TA cloning, generating the plasmid pGEM-sgBPM-N/X. This plasmid was treated with *Nde*I (NEB) and *Xho*I (NEB) to obtain the sgBPM fragments with *Nde*I and *Xho*I sticky ends, which were engineered into pET-15b to construct the bacterial expression plasmid pET-15b-sgBPM. The plasmids of pET-15b-sgBP(M) were transformed into *Escherichia coli* BL21 for protein expression. His-tagged sgBP and mutated derivatives were produced under 1 mM isopropyl-b-D-thiogalactopyranoside (IPTG) treatment when OD_600_ of transformed bacteria reached 0.4. After 24 h of incubation at 20°C and with shaking at 200 rpm, the IPTG-induced bacterial cultures were centrifuged at 6,000 x g for 10 min at 4°C, and the bacterial pellets were resuspended in 1 ml desalting buffer (50 mM Tris-Cl, 100 mM NaCl, pH 7.4). The suspended cultures were sonicated for 20 min on ice and then centrifuged at 16,000 x g for 10 min at 4°C. The supernatant of the sonicated cultures was added to His GraviTrap columns (GE Healthcare) that had been pre-equilibrated with wash buffer (20 mM imidazole, 50 mM Tris-Cl, 100 mM NaCl, pH 7.4). Subsequently, the columns were washed with 10 ml of wash buffer. The His-tagged CPs were eluted with 3 ml elution buffer (200 mM imidazole, 50 mM Tris-Cl, 100 mM NaCl, pH 7.4), followed by desalting and buffer exchange with desalting buffer via HiTrap Desalting columns (GE Healthcare). For changing pH condition, pH 5.0 citrate buffer was used under acidic conditions, and the pH 9.0 glycine-sodium hydroxide buffer was used under alkaline conditions.

### Analytical ultracentrifugation

Sedimentation velocity (SV) experiments were performed at 45,000 rpm using a 4-hole AnTi60 rotor at 20°C in a Beckman Optima XL-I AUC equipped with absorbance optics. The CP samples collected from the gel filtration column were diluted to a final concentration of 0.2, 0.5, and 0.8 mg/ml using a buffer solution of 50 mM Tris-HCl and 100 mM NaCl at pH 7.4. Standard 12-mm aluminum double-sector centerpieces were filled with protein solution, and the reference cell contained the blank buffer. Quartz windows were used with absorbance optics (OD_280_) in a continuous mode without averaging. No time interval was set between scans. The data were analyzed with a *c(s)* distribution of the Lamm equation solutions calculated by the program SEDFIT Version 14.4d (http://analyticalultracentrifugation.com). The software Sednterp (http://www.jphilo.mailway.com) was used to estimate protein partial specific volume (*Vbar*) (0.73 ml/g), buffer density (1.00379 g/ml) and buffer viscosity (0.01027 poise) at 20°C.

### Measurements of spectral properties

All spectra were measured under 25°C in a 1-cm light path quartz cuvette, and the concentrations of sgBP and its derivatives were 0.25 mg/ml. Absorption spectra ranging from 350 nm to 710 nm were measured using a Beckman DU640B spectrophotometer.

### Crystallization and data collection

Crystals of the sgBP were grown by mixing 1 μL protein of 10 mg/mL concentration with 1 μL of reservoir solution using the sitting-drop vapor diffusion method at 18°C. The sgBP crystals were obtained in a reservoir solution (24% (*w/v*) PEG 600, 0.2 M imidazole malate, pH 5.5). The crystals were flash-cooled with 20% glycerol (*v/v*) as a cryo-protectant. The diffraction data of sgBP crystals were collected at cryogenic temperatures at a wavelength of 1.5418 Å using a Rigaku FR-E+ SuperBright generator equipped with an R-AXIS HTC image-plate detector. The diffraction data were processed and scaled using the program HKL2000 [24].

### Structure determination and refinement

The crystal structure of sgBP was determined by molecular replacement using the program MOLREP of the CCP4 program suite [25], and the crystal structure of non-fluorescent CP cjBlue (PDB # 2IB5) from *Cnidopus japonicus* [9] was used as a search model. The sgBP crystals belonged to the space group *P*2_1_2_1_2_1_. Throughout the refinement, 5% of randomly selected data were set aside for cross validation with *R*_free_ values. Manual modifications of the models were performed using the program Coot [26]. Difference Fourier (Fo-Fc) maps were calculated to locate the solvent molecules. The crystal structures were refined using Refmac5 [27]. Data collection and final model statistics are shown in Table 1. The molecular figures were produced using UCSF Chimera [28]. The atomic coordinates and structure factors of sgBP have been deposited in the Protein Data Bank with accession codes PDB # 4ZB1.

**Table 1:** Data collection and refinement statistics^a^.

	sgBP
Wavelength (Å)	1.5418
Space group	*P*2_1_2_1_2_1_
Cell dimensions (Å)	*a* = 61.10, *b* = 64.77, *c* = 154.74
Resolution (Å)	25.0–2.25 (2.33–2.25)
Unique reflections	29,027
*R*_merge_ (%)	9.5 (34.5)
*I*/σ(*I*)	11.9 (3.2)
Completeness	96.6 (96.5)
Redundancy	3.6 (3.5)
**Refinement**	
Resolution (Å)	25–2.25
No. of reflections *R*_work_/*R*_free_	23,302/1,316
*R*_work_/*R*_free_	17.9/22.5
No. of atoms/Avg B factor (Å^2^)	
Protein	3,610/26.9
Water	226/33.6
PEG	22/43.3
RMSD	
Bond lengths (Å)	0.009
Bond angles (°)	1.53
Ramachandran statistics (%)^b^	
Most favored	92.3
Additionally allowed	7.7
Generously allowed	0.0
Disallowed	0.0

^a^ Values corresponding to the highest resolution shell are shown in parentheses.

^b^ The stereochemistry of the model was validated with PROCHECK.

## Results

### sgBP characterization

**Fig 1:**
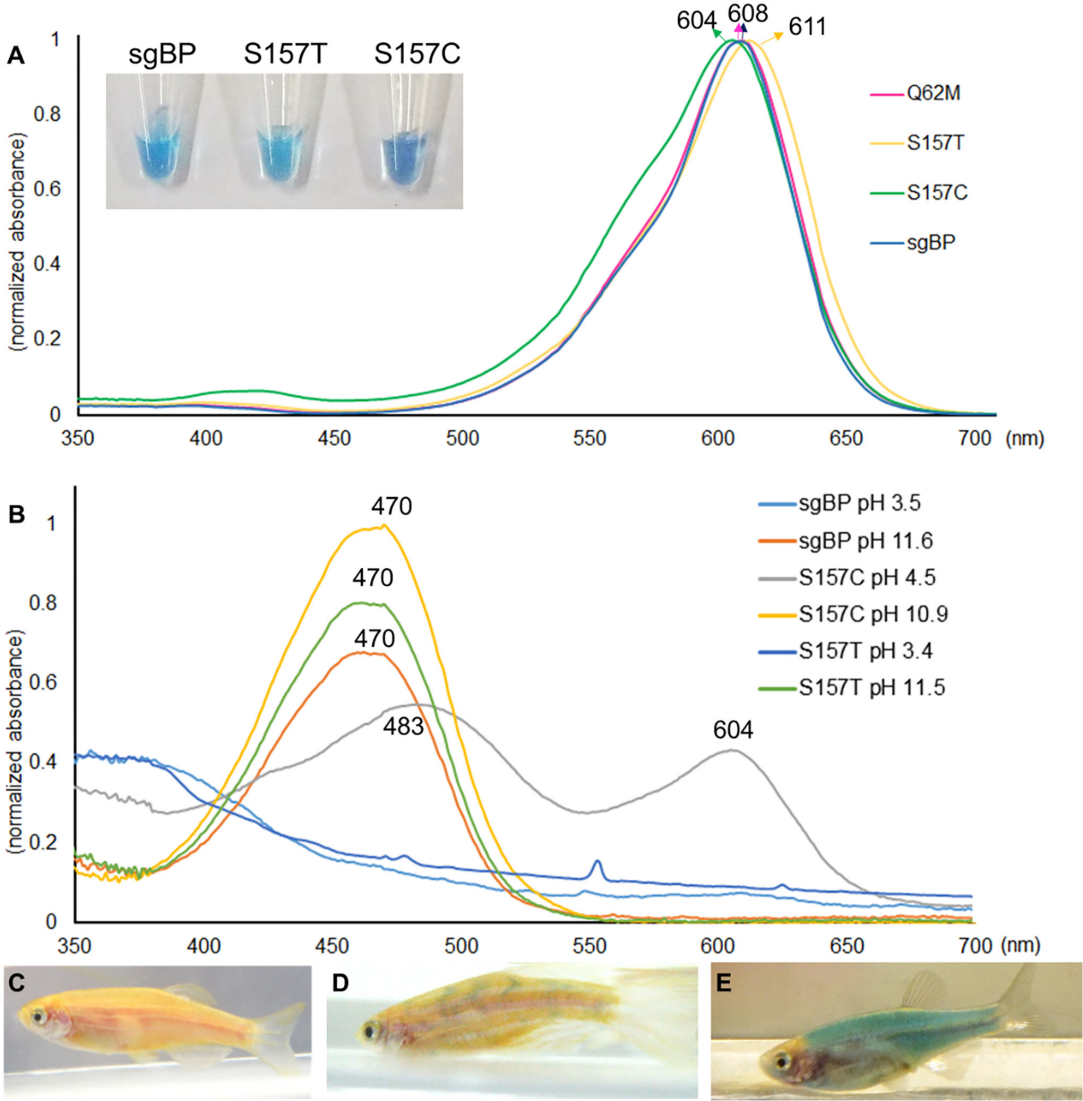
The optical properties of the sgBP protein and its mutated derivatives. (A) The absorbance spectra of sgBP and its mutated variants were measured under the concentration of 0.25 mg/ml, pH 7.4 and 25°C. The maximum absorption peaks are 608 nm for sgBP and Q62M, 611 nm for S157T and 604 nm for S157C. The colors of sgBP, S157T and S157C are blue, light blue, and deep blue, respectively. (B) The absorbance spectra of sgBP and its mutated variants change under different pH conditions. (C-E) *In vivo* expression of sgBP in zebrafish. The nontransgenic wild-type zebrafish exhibited a golden yellow appearance when they grew to 90 dpf (C), whereas the sgBP cDNA could be transiently expressed in transgenic albino zebrafish in F0 generation (D) and evenly expressed in blue in the transgenic F1 offspring (E).

**Fig 2:**
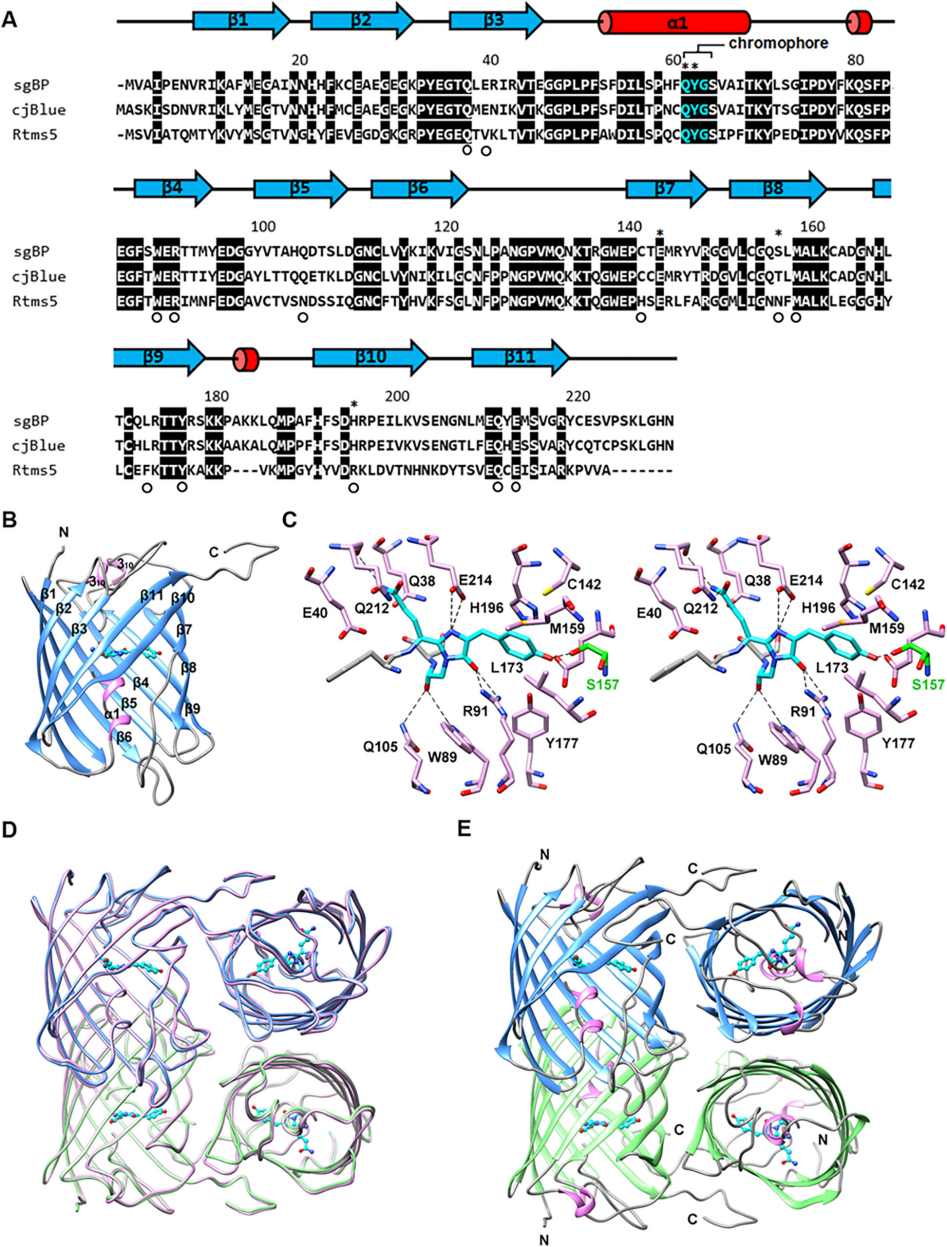
sgBP structure and chromophore environment. (A) Structure-based sequence alignment of BCPs, sgBP, cjBlue (PDB # 2IB5) and Rtms5 (PDB # 1MOU). The chromophore region is colored in cyan. The identical residues are highlighted by black boxes. The secondary structural elements according to the sgBP structure are shown. The residues in the chromophore environment are indicated by circle symbols. Residues able to alter the color characteristics are indicated by an asterisk. (B) Ribbon representation of the sgBP monomer. (C) Stereoview of the QYG chromophore environment. The QYG chromophore (cyan carbon) between F61 and S65 (with gray carbon atoms) has hydrogen bonds with W89, R91, Q105, S157 (with green carbon atoms), Q212 and E214. (D) sgBP dimer structures (purple and green ribbon) are superimposed on the tetrameric structure of cjBlue (pink ribbon). (E) The tetramer model of sgBP.

The full-length cDNA of sgBP obtained by RACE consisted of 696 bp and encoded 231 amino acid residues. The protein exhibited a blue color but did not have fluorescent properties (Fig 1A). The alignment between sgBP and other CPs in the GFP-like protein family revealed that sgBP shares 79% sequence identity with the blue CP cjBlue (Fig 2A). We then produced recombinant sgBP protein fused with an N-terminal 6xHis-tag by the pET expression system. Interestingly, the transformed bacteria turned blue when we induced them at 20°C but not 37°C, indicating that the sgBP protein could be expressed in prokaryotic cells at low temperatures (S2 Fig).

### *In vivo* expression of sgBP in zebrafish

To study whether the sgBP could be a potential biomarker, we engineered the sgBP cDNA into an expression vector containing zebrafish α-actin promoter and microinjected the expression plasmid into zebrafish. In comparison with wild-type zebrafish (Fig 1C), the sgBP could transiently exhibit in the muscle tissue of the mature transgenic fish (Fig 1D). The colors displayed in transgenic zebrafish confirmed that sgBP cDNA and its mutated sequences are faithfully expressed in higher eukaryotic organisms. Furthermore, 5 pairs of sgBP-transgenic zebrafish were selected and outcrossed individually with wild-type fish. Three sgBP-transgenic lines were generated, whose offspring ubiquitously expressed inherited sgBP in the skeletal muscle (Fig 1E).

### Overall structure of sgBP

**Fig 3:**
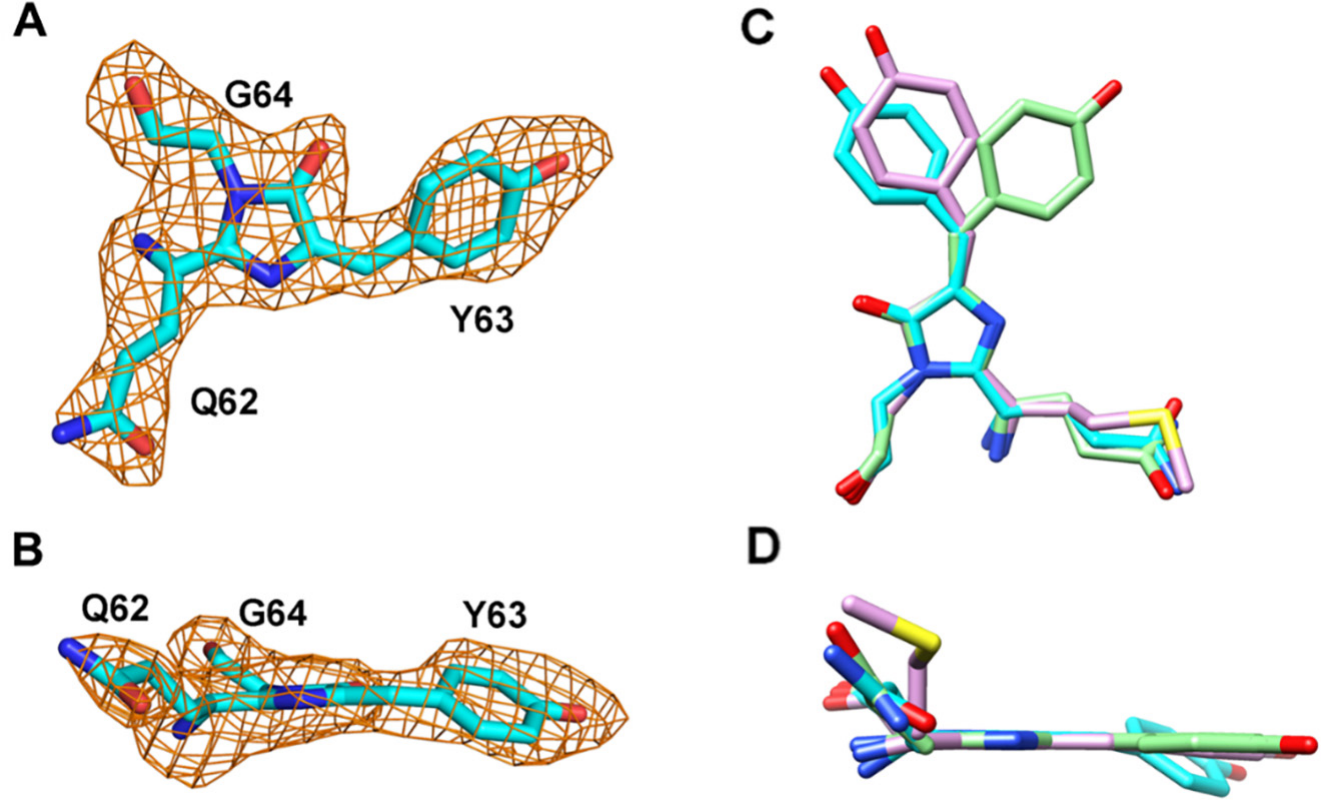
Chromophore structures and comparisons. (A, B) Fo-Fc OMIT maps (orange) were calculated for the chain A tripeptide chromophore (stick model) of sgBP and contoured at the 1.5 σ level. (C, D) Stick representations of the chromophores of sgBP, eqFP611 (PDB # 1UIS), and DsRed (PDB # 1G7K) are superimposed and colored in cyan, pink, and green, respectively. The superimposed chromophores show the *trans* non-planar conformation of the sgBP chromophore, the *trans* coplanar conformation of the eqFP611 chromophore and the *cis* coplanar conformation of DsRed chromophore.

To elucidate the structural basis of the sgBP protein and its chromophore, the recombinant sgBP (residues 1–231) with an N-terminal His-tag were purified for crystal structure determination. The sgBP was crystallized in a *P*2_1_2_1_2_1_ space group with two protein molecules in an asymmetric unit, and the final structure was refined at a resolution of 2.25 Å (Table 1). In the current structure (Fig 2B), each sgBP molecule consists of 11 β-strands with a typical 11-stranded β–can topology [11]. Two 3_10_-helices were observed on both sides of the β–can motif to form the chromophore-protecting lids. The circularized tripeptide QYG chromophore (Gln62-Tyr63-Gly64) is located at the central axis of the barrel and covalent bonds to the backbone of F61 and S65 (Fig 2C). Clear electron density for the chromophore was also observed in each protomer (Fig 3A and 3B). The C-terminal region (residues 220–231) is extended to contact with a neighboring protomer (Fig 2D and 2E). The overall structure of sgBP presented here is similar to the overall structure of cjBlue (PDB # 2IB5) [9], with an r.m.s.d. value of 0.636 Å for 225 C^α^ atoms when the monomers of two structures are superimposed. It has been proposed that natural GFP-like proteins adapt dimer or tetramer structures. In the crystal structure of sgBP (sgBP packing as a dimer), each sgBP subunit has an average surface area of 10,773 Å^2^ and an area of 1,800 Å^2^ on each protomer is buried by its counter protomer upon dimer formation. Although sgBP showed only a dimeric form in the crystal packing, the SV experiments showed that sgBP had a major sedimentation coefficient *(S)* value of 6.15, corresponding to a molecular weight of 99.5 kDa, under different concentrations. This result indicates that sgBP exists as a tetrameric state in solution (S3 Fig). For generating the tetramer model of sgBP, the dimer structures of sgBP that had been obtained were superimposed with the tetrameric state of cjBlue (PDB # 2IB5) [9] (Fig 2D and 2E).

### Chromophore structure and environment

The sgBP chromophore is derived from the tripeptide Gln-Tyr-Gly, forming a 5-[(4-hydroxyphenyl)methylene]-imidazolinone group (Fig 3A and 3B). The side chain atoms of Q62 were hydrogen-bonded to the side chain N atom and side chain O atom of Q212, which stabilized the glutaminyl group of the chromophore. The imidazolinone group is derived from the autocyclization of Q62 and G64. The cyclization step is catalyzed by the amino acids of R91 and E214, corresponding to the amino acids of R96 and E222 in avGFP [15, 16].

The imidazolinone group, forming a π-conjugation system with the phenolic group of Y63, contributes to the absorption spectrum of sgBP. The imidazolinone group interacted with R91 and E214 via hydrogen bonds. The backbone oxygen of G64 creates hydrogen bonds to the side chains of W89 and Q105, which provide the stabilizing force to the G64 end of the chromophore. The phenolic group of Y63 plays an important role in excited state proton transfer (ESPT), which determines the absorption spectrum. Y63 stacks with the side chains of M159, L173 and H196, and the side chain of Y63 makes a strong hydrogen-bond interaction of distance = 2.6 Å with the side chain of S157 (Fig 2C). The chromophore conformation and the phenolic group orientation were determined to be a *trans* non-coplanar configuration by the surrounding stacking force and hydrogen bonds. In comparison with DsRed and eqFP611, the *trans* non-coplanar conformation of the sgBP chromophore (Fig 3C and 3D) resulted in the non-fluorescent property.

### Mutations of sgBP

According to the crystal structure, several mutations have been conducted on sgBP to examine the color formation mechanism, and the results are listed in Table 2. The chromophore composition of the GFP-like protein family is usually X-Tyr-Gly. The first two residues of the chromophore are variable, whereas the third residue (Gly) is conserved [29]. According to the chromophore composition of existing FPs and CPs such as Ser-Tyr-Gly in GFP, Met-Tyr-Gly in aeCP597, and Glu-Tyr-Gly in shCP [8, 30, 31], we substituted the first residue of the sgBP chromophore on different amino acids. Gln62 residue was substituted with Ser (Q62S), Met (Q62M) and Gln (Q62E). The Q62M mutated protein remains the original color, whereas the Q62S and Q62E turn to a colorless protein (data not shown). In the cjBlue protein, the Y64L substitution proved that the stacking force between Y64 and H197 is responsible for stabilizing the *trans* chromophore conformation [9].

**Table 2:** Summary of mutation sites on sgBP.

Site	Mutation	λ_max_ (nm)	ε (M^-1^ cm^-1^)	Description
Wild type	None	608	122,573	blue
Residues in chromophore				
Gln62	Q62S	-	-	colorless
	Q62M	608	122,923	blue
	Q62E	-	-	colorless
Tyr63	Y63L	-	-	colorless
	Y63F	-	-	colorless
Residues around chromophore				
Glu144	E144D	-	-	colorless
	E144R	-	-	colorless
Ser157	S157T	611	85,654	light blue
	S157C	604	58,029	dark blue
His196	H196Y	-	-	colorless
	H196A	-	-	colorless
Ser157+His196	S157T/H196R	-	-	colorless
	S157C/H196R	-	-	colorless

The Y63L and Y63F mutation in sgBP were used to examine the interaction between the phenolic group in the chromophore and the surrounding environment. However, the Y63L and Y63F mutant proteins both turned colorless. Several residues around the phenolic group of the chromophore were also chosen for altering the configuration of the chromophore based on the crystal structure of sgBP. The E144 was substituted with Asp and Arg, and both substitutions led to colorless mutants (data not shown). The H196 in sgBP was substituted with Ala and Tyr for changing the stacking effect with the side chain of Y63. The H196A and H196Y were turned into colorless proteins (data not shown) and showed that the alteration of H196 would affect color generation in sgBP. These results suggested that the blue color determination is related to the interaction between Y63 of the chromophore and the surrounding residues. The S157 was substituted for Thr and Cys (with a thiol side chain pK_a_ = 8.37) to alter the chromophore environment. The mutated S157T protein exhibited a light blue color, and the S157C protein exhibited a deep blue color (Fig 1A). The color alteration indicated that the interaction between the phenolic group in the chromophore and the side chain of 157 is essential for color determination.

### Optical properties of sgBP and derivatives

The sgBP and its derivatives were taken to examine the optical properties. The original absorption spectrum of sgBP showed a large absorbance of yellow and red light and has a λ_max_ at 608 nm (Fig 1B), resulting in the blue color. The ε of sgBP at 608 nm is 122,573 M^-1^ cm^-1^. Substitutions in the chromophore region sometimes did not cause a major change in the photo properties. For example, the Q62M mutation showed an absorption spectrum nearly identical to sgBP (Fig 1B) and had an ε of 122,923 M^-1^ cm^-1^. The mutation at S157 in the chromophore environment had the ability to alter the absorption spectrum. The S157T mutation shifts the absorbance spectrum toward the red light region, and the λ_max_ of the S157T mutated protein is located at 611 nm (Fig 1B). The ε of S157T at 611 nm is 85,654 M^-1^ cm^-1^. Another mutation at the S157 site, S157C, shifts the absorbance toward the blue region, and the λ_max_ of the S157C mutated protein is located at 604 nm (Fig 1B). The ε of S157C at 604 nm is 58,029 M^-1^ cm^-1^.

### pH effects on sgBP and derivatives

The optical properties of the sgBP, S157T and S157C mutations at different pH values were measured (Fig 1B). The λ_max_ of sgBP remained at 608 nm in the pH range of 3.5–11.6, but the total absorption decreased in weak acidic (approximately pH 4.5) and weak alkaline (approximately pH 9.5) solutions (S4 Fig). When the environment is out of the tolerance range, sgBP would turn to a yellow aggregation, with a λ_max_ = 470 nm, at pH>11.6 and would be colorless at pH<3.5. Similar characteristics are exhibited by S157T and S157C mutated proteins. In comparison with sgBP, the pH tolerance of S157T and S157C ranged from pH 3.4–11.5 and pH 4.5–10.9, respectively. Both mutated proteins had a shifted λ_max_ approximately 470 nm when in the alkaline solution. In an acidic environment, S157T exhibited colorless characteristics similar to sgBP, but S157C would exhibit a colorless form with two peaks, 483 nm and 608 nm, respectively. Additionally, both the yellow aggregation and the colorless form could not be recovered by changing the pH value to neutral.

## Discussion

### Chromophore mutations affect sgBP color expression

The Gln-Tyr-Gly tripeptide chromophore of sgBP is similar to other BCPs such as Rtms5 and cjBlue (Fig 2A) [7, 9]. Mutations at the first two amino acids of the chromophore usually shift the maximum excitation and emission peak of GFP-like proteins, accompanied by changes in the optical properties [30–33]. We used the blue aeCP597 [8] and the fluorescent GFP chromophore composition [30] as the references to conduct mutagenesis on the sgBP chromophore. In our results, the Q62M mutation did not change the pattern of total absorption spectrum. The absorption spectrum and the ε of the Q62M mutant were nearly identical to the sgBP (Fig 1B). The Q62S mutation resulted in the loss of the blue color characteristic. These results indicated that the substitution of the chromophore Gln may either retain the original optical properties or lose the optical properties and implied that Q62 was not a good mutation site for changing the color expression of sgBP.

### Mutations in the chromophore environment

The orientation and conformation of the phenolic group in the chromophore play important roles in determining the optical properties of GFP-like proteins. Hence, the Y63L mutation was conducted on the sgBP chromophore. However, the Y63L mutation resulted in the loss of the blue color and without the addition of another color or generation of fluorescence. The residues around the phenolic group of the sgBP chromophore are composed of C142, E144, S157, M159, L173, Y177 and H196 (Fig 2C). Two residues, the E144 and H196, were chosen for mutation because of their positions relative to the phenolic group. The E144D, E144R, H196A and H196Y mutations turned the mutated protein colorless. And the colorless characteristic is not caused by improper expression of the mutated proteins in *E*. *coli*. Take the E144 mutated proteins for example, the mutated proteins are colorless and have the same molecular weight as original sgBP (S5 Fig). The results suggested that a single mutation in the environment around the phenolic group of Y63 may change the *cis-trans* configuration and the coplanarity of the chromophore and lead to the loss of the color properties.

### The effects of S157 mutations

According to the crystal structure of sgBP, the hydroxyl group of the phenolic moiety has a hydrogen bond interaction with the side chain of S157 at distance of 2.6 Å (Fig 2C). We suggest that this hydrogen bond is involved in the conjugated system and may affect the absorption spectrum of sgBP. The colorless mutant Y63F protein showed that the hydroxyl group is essential for color formation. The S157 and C142 of sgBP, corresponding to the F165 and H148 of GFP, are key sites for regulating the quantum yield in GFP-like proteins [2]. To test whether the interaction between the Y63 in the chromophore and S157 could modify the absorption spectrum, the S157 was replaced by Thr, identical to the T158 in cjBlue and possessing similar properties to Ser and Cys, having a thiol side chain with pK_a_ of 8.37. Interestingly, the color of the S157C mutant protein turned to deep blue, the maximum absorption peak shifted to 604 nm, and the absorbance of the overall absorption spectrum is lower than the original sgBP with an ε of 58,029 M^-1^ cm^-1^. We suggest that the peak shifts are due to the deprotonation of the phenolic group caused by C157. Because the side chain pK_a_ value of the thiol group (8.37) of Cys is lower than the side chain pK_a_ value of the phenolic group (10.46) of the chromophore, the thiol group would tend to dissociate, forming a negative charged thiol group like cysteine protease [34], and the phenolic group is likely to be partially deprotonated by the negative charged thiol group of Cys. The electronic conjugation system between the imidazolinone ring and the phenolic group may be changed and could absorb the light that has shorter wavelengths than the original 608 nm absorption peak in the sgBP absorption spectrum.

### pH effects on sgBP and S157 mutated proteins

The optical properties of sgBP and S157 mutated proteins are relatively stable. In the pH tolerance range of sgBP (pH 3.5–11.6) (S4 Fig), the λ_max_ of sgBP remains unchanged and only the total absorbance would be affected, so the color of sgBP could be maintained. When the pH value is out of the tolerance range, sgBP would start turning to a yellow aggregation under alkaline condition, pH>11.6, and the sgBP would become colorless under acidic pH<3.5 conditions. Both transitions are irreversible. Similar characteristics are exhibited by S157 mutations. The S157T is stable in the pH range of 3.4–11.5, and the S157C is stable in the pH range of 4.5–10.9. These results show that the S157 mutations could also change the pH tolerance of the protein. The pH value changed from pH 4.5 to pH 9.5 under buffered conditions may affect only the protein surface environment, corresponding to the variation of total absorbance, and the unchanged λ_max_ indicates that the chromophore group shows a stable conformation in the pH tolerance range.

### The deprotonation effect in sgBP

**Fig 4:**
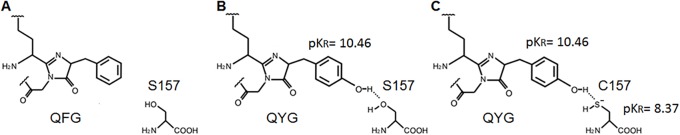
Putative mechanism of S157 site alters the deprotonation state on the phenolic group of the chromophore. Three different deprotonation states on the hydroxyl group of the phenolic region in the sgBP chromophore. (A) S157 with mutated QFG chromophore; (B) S157 with natural QYG chromophore; (C) Mutated C157 with QYG chromophore. The chromophore encounters a stronger deprotonation effect by the thiol group.

In general, the protonation-deprotonation state of the phenolic group of the chromophore could affect the ESPT, resulting in the shift of the absorption spectrum and sometimes making the protein photoswitchable [35]. In the photoswitchable rsTagRFP, the protonation-deprotonation states of the phenolic group in the chromophore switch the protein between the *trans* (protonated, off-state) and *cis* form (deprotonated, on-state) [36]. Based on the stacking effect between the H196 and the Y63 site [9] and the sequence alignment of cjBlue and Rtms5 (Fig 2A), we tried to combine the H196R according to the Rtms5 sequence with S157T and S157C mutations to enhance the deprotonation effect. However, the color of mutated sgBP disappeared, indicating that the deprotonation effect in S157 mutants would be affected by the His196 site. The alignment results showed that the mutated sites in sgBP are nearly identical to the cjBlue sequence except for the 157 site (Fig 2A). When the S157T mutation was applied to the sgBP sequence, the λ_max_ of S157T was shifted to 611 nm, the same as the λ_max_ of cjBlue [9]. By comparison, the S157C mutation shifted the λ_max_ to a shorter wavelength, 604 nm. The wavelength shift changed the reflection mixture of light after its absorption, which determines the color of a chromoprotein. These results suggest that S157 is important for blue color generation and also indicate that the deprotonation state of the phenolic group in the chromophore could be controlled by S157, which may change the λ_max_ and regulate the color expression of the mutated protein (Fig 4).

### sgBP as a biomarker

Upon expressing sgBP in zebrafish driven by the α-actin promoter, the expressed sgBP could be observed (Fig 1D and 1E). The expression of sgBP in zebrafish muscle cells suggests that the sgBP could fold and express properly in the intracellular pH range (approximately 7.55) [37] because the intracellular pH range of living cells is included in the pH tolerances of sgBP and its derivatives. On the basis of *in vitro* and *in vivo* chromoprotein expressions, we suggest that sgBP is a valuable biomarker. The sgBP can be expressed correctly in other organisms, and the color of sgBP can be observed by the naked eye. The dimeric sgBP crystal structure also provides the possibility of sgBP to be engineered to a more applicable dimer form.

## Conclusions

Both cnidarian CPs and FPs could serve as potential biomarkers. However, the naturally existing blue color exists in only CPs but not FPs. High-energy short wavelength light ranging from ultraviolet to green, which may be harmful to organisms, is needed for the excitation of BFPs to emit fluorescence, but CPs have the advantage that they can be observed under visual light. In this study, the identification of sgBP provides a new choice for a blue biomarker. The crystal structure of sgBP demonstrates the engineering potential of the dimer form. Structure-based mutations applied to sgBP reveal the sites affecting the blue color generation. Additionally, the λ_max_ of sgBP could be regulated by a mutation at S157 through altering the deprotonated state of the phenolic group in the chromophore. These results provide a structural basis for the blue color enhancement of the biomarker development.

## Supporting Information

S1 Fig Peptide mapping of blue chromoprotein of *S*. *gigantea*. The typsin-digested blue CP major band of crude protein extracts was specifically analyzed by LC-MS/MS. The partial peptide sequences of sgBP were shown, and the peptides matching the chromoprotein of *A*. *equina* are presented in bold red.
(DOCX)

S2 Fig Expression of sgBP at different temperatures. The expression of sgBP protein in *E*. *coli* under 20°C and 30°C. After 24 hours of incubation, the sgBP was expressed and could be pelleted under 20°C although the sgBP could not be observed under 37°C.
(DOCX)

S3 Fig Analytical ultracentrifugation results of sgBP. The sedimentation coefficient distribution profiles of the different sgBP concentrations of 0.2, 0.5, and 0.8 mg/ml. The predicted sedimentation coefficient by SEDFIT is 6.15, corresponding to 99.3 kDa, which indicates the tetramer form of sgBP.
(DOCX)

S4 Fig Absorption spectra of sgBP at different pH values in the pH tolerance range. The absorbance spectra of sgBP were measured at the concentration of 0.25 mg/ml and at 25°C, with different environmental pH values of 4.5, 5.5, 6.5, 8.5 and 9.5.
(DOCX)

S5 Fig The colorless E144 mutated proteins are expressed in *E*. *coli*. (A) The sgBP-E144D and sgBP-E144R mutated protein are colorless when expressed. (B) The colorless crude protein extractions are analyzed on a 12% SDS-PAGE. Arrowhead indicates that the colorless mutated proteins have similar molecular weight to original sgBP. M: molecular markers; NI144D, NI144R: non-induced E144D and E144R bacterial extraction as control. E144D, E144R: The colorless crude protein extractions of sgBP-E144D and sgBP-E144R expressed bacteria.
(DOCX)
