# Supplementary material for: Chromophore Deprotonation State Alters the Optical Properties of Blue Chromoprotein
Source: PLoS One. 2015 Jul 28;10(7):e0134108. doi: 10.1371/journal.pone.0134108 (PMC4517874; doi:10.1371/journal.pone.0134108)
Supplement: S1 Fig — The typsin-digested blue CP major band of crude protein extracts was specifically analyzed by LC-MS/MS. The partial peptide sequences of sgBP were shown, and the peptides matching the chromoprotein of A. equina are presented in bold red. (DOCX) [file pone.0134108.s001.docx]

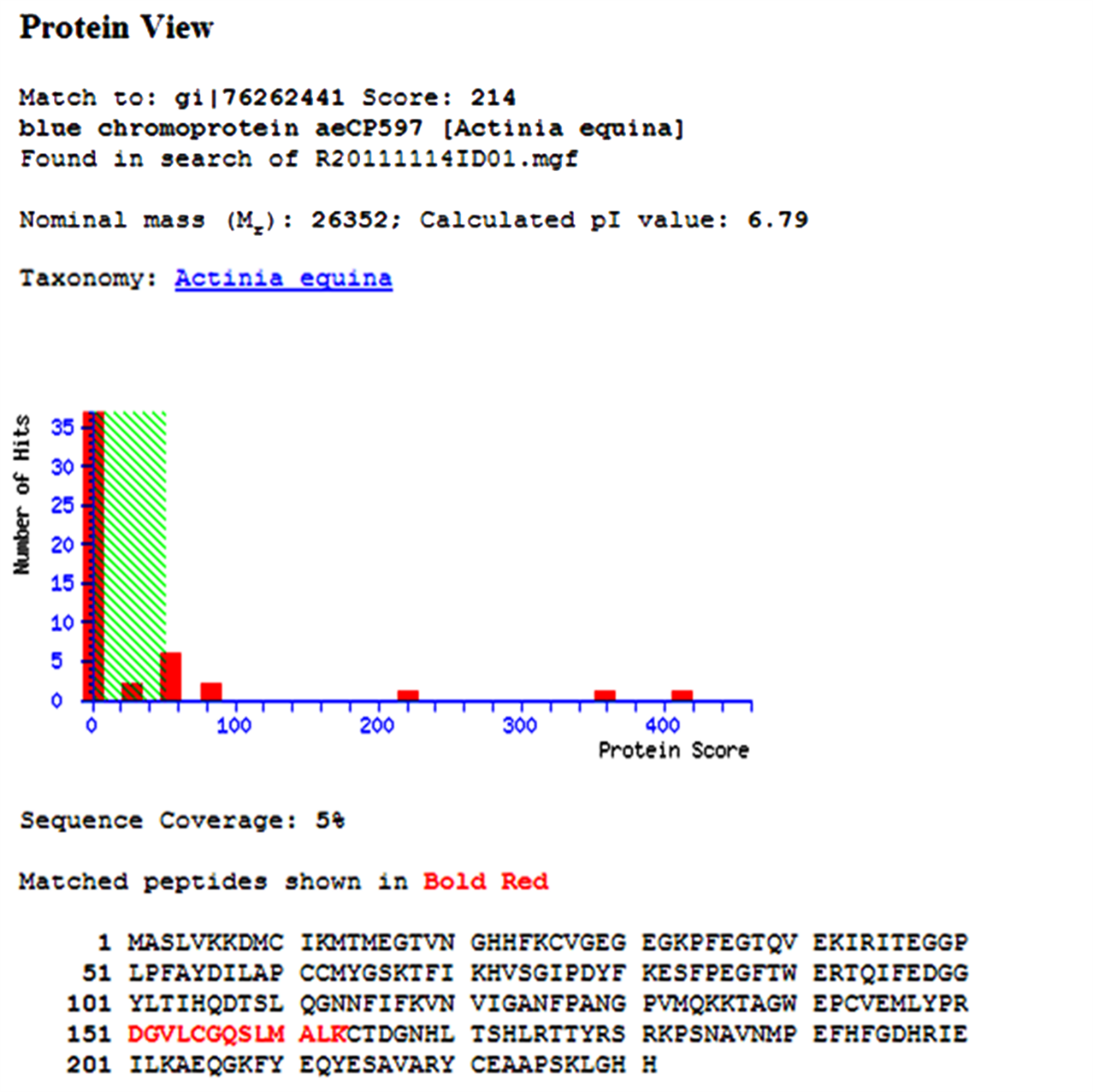


**S1 Fig. Peptide mapping of blue chromoprotein of *S. gigantea*.** The typsin-digested blue CP major band of crude protein extracts was specifically analyzed by LC-MS/MS. The partial peptide sequences of sgBP were shown, and the peptides matching the chromoprotein of *A. equina* are presented in bold red.
