# Supplementary material for: Chromophore Deprotonation State Alters the Optical Properties of Blue Chromoprotein
Source: PLoS One. 2015 Jul 28;10(7):e0134108. doi: 10.1371/journal.pone.0134108 (PMC4517874; doi:10.1371/journal.pone.0134108)
Supplement: S2 Fig — The expression of sgBP protein in E. coli under 20°C and 30°C. After 24 hours of incubation, the sgBP was expressed and could be pelleted under 20°C although the sgBP could not be observed under 37°C. (DOCX) [file pone.0134108.s002.docx]

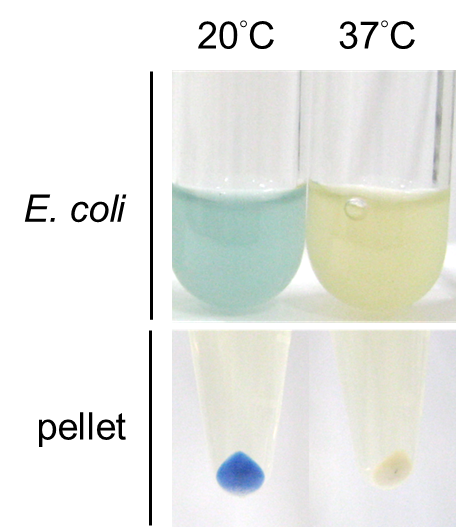


**S2 Fig. Expression of sgBP at different temperatures.** The expression of sgBP protein in *E. coli* under 20°C and 30°C. After 24 hours of incubation, the sgBP was expressed and could be pelleted under 20°C although the sgBP could not be observed under 37°C.
