# Supplementary material for: Chromophore Deprotonation State Alters the Optical Properties of Blue Chromoprotein
Source: PLoS One. 2015 Jul 28;10(7):e0134108. doi: 10.1371/journal.pone.0134108 (PMC4517874; doi:10.1371/journal.pone.0134108)
Supplement: S3 Fig — The sedimentation coefficient distribution profiles of the different sgBP concentrations of 0.2, 0.5, and 0.8 mg/ml. The predicted sedimentation coefficient by SEDFIT is 6.15, corresponding to 99.3 kDa, which indicates the tetramer form of sgBP. (DOCX) [file pone.0134108.s003.docx]

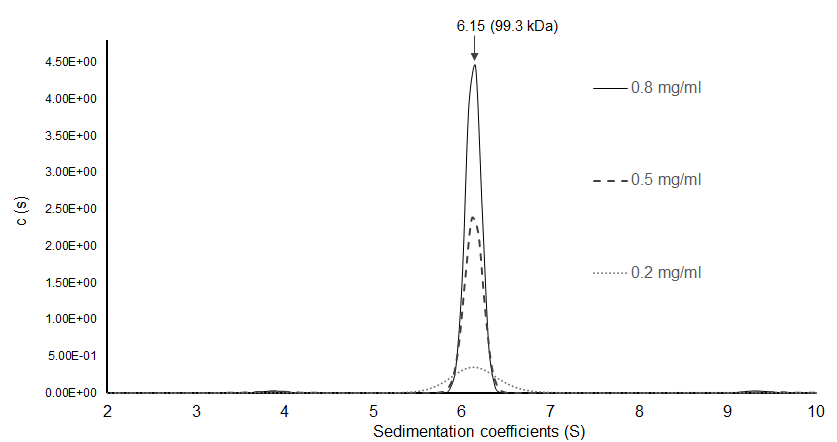


**S3 Fig.** **Analytical ultracentrifugation results of sgBP.** The sedimentation coefficient distribution profiles of the different sgBP concentrations of 0.2, 0.5, and 0.8 mg/ml. The predicted sedimentation coefficient by SEDFIT is 6.15, corresponding to 99.3 kDa, which indicates the tetramer form of sgBP.
