# Supplementary material for: Chromophore Deprotonation State Alters the Optical Properties of Blue Chromoprotein
Source: PLoS One. 2015 Jul 28;10(7):e0134108. doi: 10.1371/journal.pone.0134108 (PMC4517874; doi:10.1371/journal.pone.0134108)
Supplement: S4 Fig — The absorbance spectra of sgBP were measured at the concentration of 0.25 mg/ml and at 25°C, with different environmental pH values of 4.5, 5.5, 6.5, 8.5 and 9.5. (DOCX) [file pone.0134108.s004.docx]

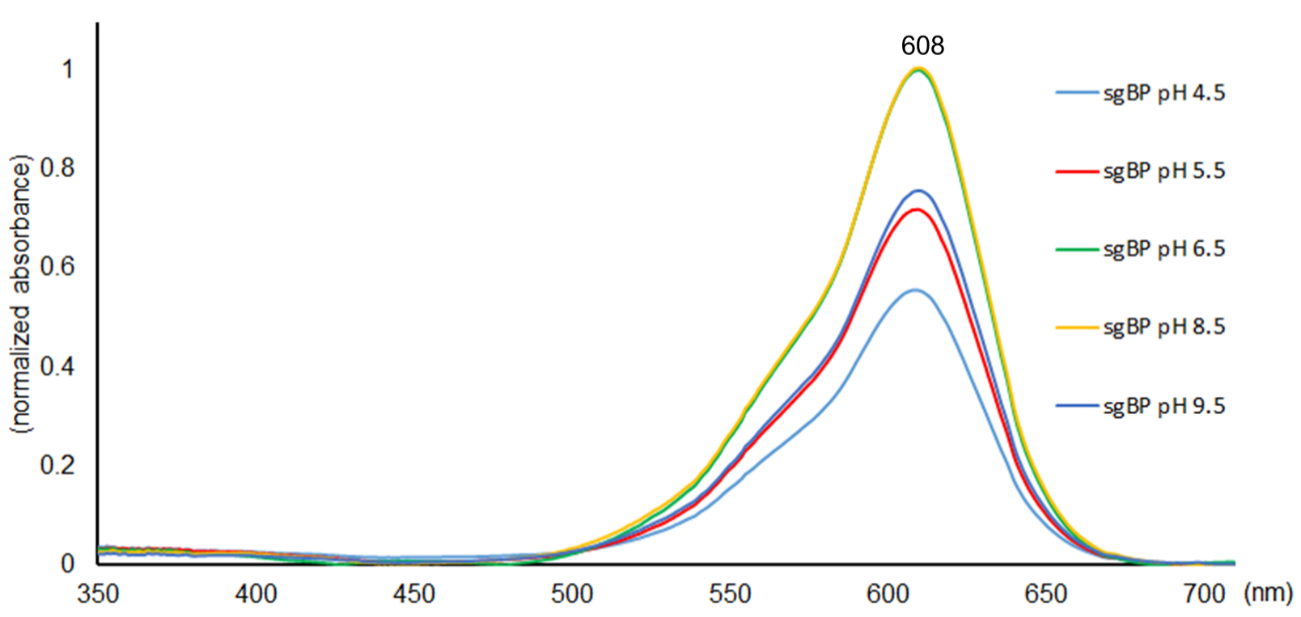


**S4 Fig.** **Absorption spectra of sgBP at different pH values.** The absorbance spectra of sgBP were measured at the concentration of 0.25 mg/ml and at 25°C, with different environmental pH values of 4.5, 5.5, 6.5, 8.5 and 9.5.
