# Supplementary material for: Chromophore Deprotonation State Alters the Optical Properties of Blue Chromoprotein
Source: PLoS One. 2015 Jul 28;10(7):e0134108. doi: 10.1371/journal.pone.0134108 (PMC4517874; doi:10.1371/journal.pone.0134108)
Supplement: S5 Fig — (A) The sgBP-E144D and sgBP-E144R mutated protein are colorless when expressed. (B) The colorless crude protein extractions are analyzed on a 12% SDS-PAGE. Arrowhead indicates that the colorless mutated proteins have similar molecular weight to original sgBP. M: molecular markers; NI144D, NI144R: non-induced E144D and E144R bacterial extraction as control. E144D, E144R: The colorless crude protein extractions of sgBP-E144D and sgBP-E144R expressed bacteria. (DOCX) [file pone.0134108.s005.docx]

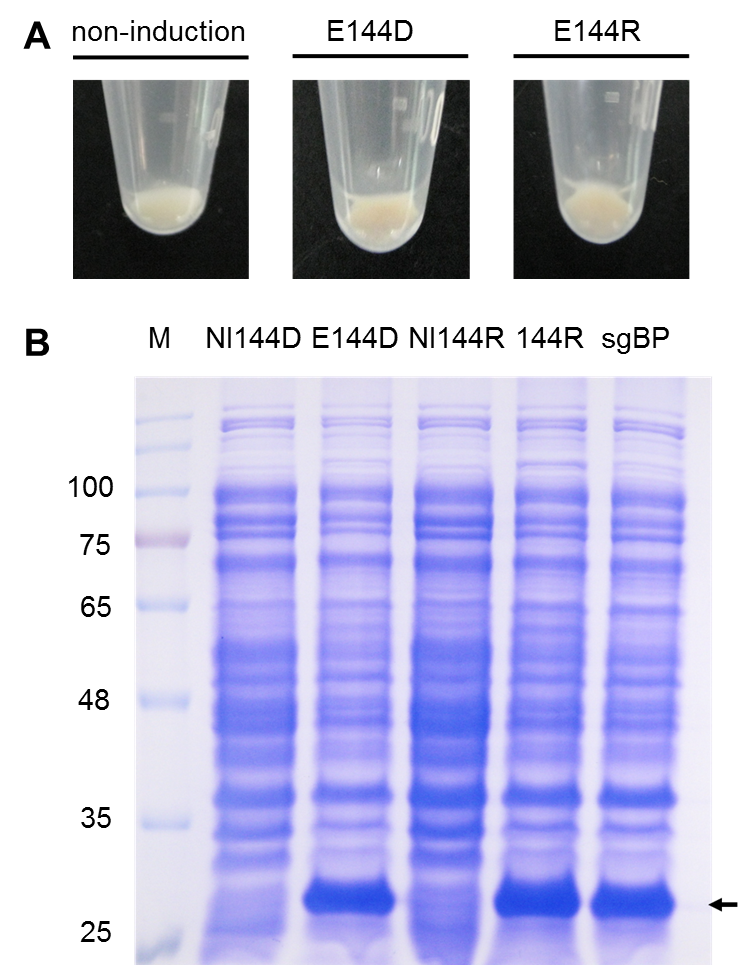


**S5 Fig.** **The colorless E144 mutated proteins are expressed in *E. coli*.** (A) The sgBP-E144D and sgBP-E144R mutated protein are colorless when expressed. (B) The colorless crude protein extractions are analyzed on a 12% SDS-PAGE. Arrowhead indicates that the colorless mutated proteins have similar molecular weight to original sgBP. M: molecular markers; NI144D, NI144R: non-induced E144D and E144R bacterial extraction as control. E144D, E144R: The colorless crude protein extractions of sgBP-E144D and sgBP-E144R expressed bacteria.
